# Additive Effect between IL-13 Polymorphism and Cesarean Section Delivery/Prenatal Antibiotics Use on Atopic Dermatitis: A Birth Cohort Study (COCOA)

**DOI:** 10.1371/journal.pone.0096603

**Published:** 2014-05-21

**Authors:** So-Yeon Lee, Jinho Yu, Kang-Mo Ahn, Kyung Won Kim, Youn Ho Shin, Kyung-shin Lee, Seo Ah Hong, Young-ho Jung, Eun Lee, Song-I Yang, Ju-hee Seo, Ji-Won Kwon, Byoung-Ju Kim, Hyo-Bin Kim, Woo-Kyung Kim, Dae Jin Song, Gwang Cheon Jang, Jung Yeon Shim, Soo-Young Lee, Ja-Young Kwon, Suk-Joo Choi, Kyung-Ju Lee, Hee Jin Park, Hye-Sung Won, Ho-Sung Yoo, Mi-Jin Kang, Hyung-Young Kim, Soo-Jong Hong

**Affiliations:** 1 Department of Pediatrics, Hallym University College of Medicine, Anyang, Korea; 2 Childhood Asthma Atopy Center, Asan Medical Center, Seoul, Korea; 3 Department of Pediatrics, Sungkyunkwan University of School of Medicine, Seoul, Korea; 4 Department of Pediatrics, College of Medicine, Yonsei University, Seoul, Korea; 5 Department of Pediatrics, CHA University of School of Medicine, Seoul, Korea; 6 The Asan Institute for Life Science, Seoul, Korea; 7 Department of Pediatrics, Korea Cancer Center Hospital, Seoul, Korea; 8 Department of Pediatrics, Seoul National University Bundang Hospital, Seongnam, Korea; 9 Department of Pediatrics, Inje University College of Medicine, Seoul, Korea; 10 Department of Pediatrics, Korea University Guro Hospital, Seoul, Korea; 11 Department of Pediatrics, National Health Insurance Corporation Ilsan Hospital, Goyang, Korea; 12 Department of Pediatrics, Ajou University School of Medicine, Suwon, Korea; 13 Department of Obstetrics and Gynecology, Yonsei University College of Medicine, Seoul, Korea; 14 Department of Obstetrics and Gynecology, Samsung Medical Center, Sungkyunkwan University School of Medicine, Seoul, Korea; 15 Department of Obstetrics and Gynecology, Pochon CHA University College of Medicine, Seoul, Korea; 16 Department of Obstetrics and Gynecology, Asan Medical Center, University of Ulsan College of Medicine, Seoul, Korea; 17 Department of Pediatrics, Kosin University College of Medicine, Busan, Korea; Université Libre de Bruxelles, Belgium

## Abstract

**Background:**

Although cesarean delivery and prenatal exposure to antibiotics are likely to affect the gut microbiome in infancy, their effect on the development of atopic dermatitis (AD) in infancy is unclear. The influence of individual genotypes on these relationships is also unclear. To evaluate with a prospective birth cohort study whether cesarean section, prenatal exposure to antibiotics, and susceptible genotypes act additively to promote the development of AD in infancy.

**Methods:**

The Cohort for Childhood of Asthma and Allergic Diseases (COCOA) was selected from the general Korean population. A pediatric allergist assessed 412 infants for the presence of AD at 1 year of age. Their cord blood DNA was subjected to interleukin (IL)-13 (rs20541) and cluster-of-differentiation (CD)14 (rs2569190) genotype analysis.

**Results:**

The combination of cesarean delivery and prenatal exposure to antibiotics associated significantly and positively with AD (adjusted odds ratio, 5.70; 95% CI, 1.19–27.3). The association between cesarean delivery and AD was significantly modified by parental history of allergic diseases or risk-associated IL-13 (rs20541) and CD14 (rs2569190) genotypes. There was a trend of interaction between IL-13 (rs20541) and delivery mode with respect to the subsequent risk of AD. (*P* for interaction = 0.039) Infants who were exposed prenatally to antibiotics and were born by cesarean delivery had a lower total microbiota diversity in stool samples at 6 months of age than the control group. As the number of these risk factors increased, the AD risk rose (trend p<0.05).

**Conclusion:**

Cesarean delivery and prenatal antibiotic exposure may affect the gut microbiota, which may in turn influence the risk of AD in infants. These relationships may be shaped by the genetic predisposition.

## Introduction

Although cesarean delivery and prenatal exposure to antibiotics are likely to affect the gut microbiome in infancy [1], [2], their effect on the development of atopic dermatitis (AD) in infancy is unclear [3]–[6].

Microbes are recognized by the innate immune system using pattern recognition receptors (PRRs). cluster-of-differentiation (CD)14 is, together with Toll-like receptor(TLR)4, involve in the recognition and signal transduction of bacterial endotoxin, a major component of the bacterial cell wall of gram negative bacteria. Downstream affects of CD14/TLR receptor activation on antigen presenting cells include the release of cytokines, such as IL-10, and IL-12 [7]. Interleukin (IL)-13 is a cytokine typically produced during Th2 responses and plays a crucial role in atopy and allergic diseases [8].

Several studies show that polymorphisms in the immune system-related genes IL-13 and CD14 associate with AD [9]–[11]. However, the results from cross-sectional and case-control studies on the influence of these gene polymorphisms on the development of AD in different populations are inconsistent [12]–[14]. They may reflect differences between the studies in terms of environmental factors that modify genetic associations.

Early microbial contact (i.e., during fetal life and infancy) has been suggested to be involved in the initiation and perpetuation of the aberrant immune activation and responsiveness that plays a central role in the pathogenesis of allergic diseases. This may be particularly true for AD, which appears early in life. Individual immune system-related genotypes may also shape the responsiveness to gut microbes during fetal life and infancy.

It was hypothesized that prenatal exposure to antibiotics and cesarean delivery may affect the gut microbiota in early infancy, which is an important period in the development of the immune system. It was also hypothesized that these prenatal risk factors may be modified by the genetic background. This is the first study to assess how cesarean section birth, prenatal antibiotic exposure, and IL-13 and CD14 risk alleles interact in the development of AD in infancy.

## Methods

### Ethics Statement

This study was approved by the institutional review board of the Asan Medical Center (IRB No. 2008-0616), the Samsung Medical Center (IRB No. 2009-02-021), the Severance Hospital (IRB No. 4-2008-0588) and the CHA Medical Center (IRB No. 2010-010). Written informed consent was confirmed by each IRB and obtained from the parents of each infant.

### Study Population

The Cohort for Childhood Origin of Asthma and Allergic Diseases (COCOA) was composed of the general Korean population after recruiting healthy pregnant women who delivered at four hospitals in a metropolitan city (Seoul). The recruitment period commenced in December 2007. A modified questionnaire of the International Study of Asthma and Allergies in Childhood (ISAAC) was completed by the parents at 36 weeks gestational age [15]. The delivery mode and other prenatal variables were extracted from the maternal and neonatal medical records shortly after delivery. The presence of AD was clinically diagnosed by pediatric allergy specialists using the criteria of Hanifin and Rajka [16] when the infants were followed up at the hospital at 12 months of age.

Of the 534 12-month infants who were enrolled, 122 infants dropped out. At the time the present study was conducted, 412 of the infants met the inclusion criteria: age>1 year, examined for the presence of AD at 12 months by pediatric allergy specialists, and completion of the 12 month questionnaire by the parents.

### DNA Collection and SNP Genotyping

Genomic DNA was extracted from the cord blood mononuclear cells of each child and genotyped for the IL-13 rs2569190 and CD14 rs1927911 polymorphisms by using the TagMan fluorogenic 5′ nuclease assay (ABI, Foster City, CA, USA). The endpoint fluorescent readings were performed on an ABI 7900 HT Sequence Detection System (ABI, Foster City, CA, USA). Duplicate samples and negative controls were included to ensure genotyping accuracy.

### Microbial Analysis

Fecal samples were obtained from 11 of the 412 infants in this study at the age of 6 months. Of these, six had both prenatal antibiotic exposure and were delivered by cesarean section, while the remaining five were delivered vaginally and were not exposed to antibiotics prenatally. These infants were selected randomly. The fecal sampling and DNA extraction methods have been described in detail elsewhere [17].

### Statistical Analysis

The means and standard deviations or frequencies and percentages of general characteristics were presented for overall study population.

The multivariable logistic regression models were used to estimate odd ratios (OR) and corresponding 95% confidence intervals (95% CI) for comparison of AD at infancy occurrence risk by relevant covariates. The following covariates were considered as potential confounders: gestational age at birth, sex, pre-pregnancy maternal body-mass index (BMI), maternal age at delivery, maternal education level, prenatal exposure to smoke, prenatal exposure to pets, presence of older siblings, and parental allergic disease history. The regression models included *a priori* potential confounding co-variables which are known risk factors of infant AD established in the literature, and significant variables identified from initial univariate analyses (p<0.2). Data from subjects were divided into 4 groups based on environmental factors (delivery mode and prenatal exposure to antibiotics) and genetic background (parental history of allergic diseases and genotypes) and logistic regression were used to estimate aORs.

Finally, we tested for trends regarding the effect of the number of risk factors (early life environmental factors and genetic polymorphisms) on the development of AD in infancy. P-value for trends was determined using the general linear model for continuous variables.

All statistical tests were two-sided significance levels of p value less than 0.05. Statistical analyses were done with IBM SPSS Statistics 20.0 (IBM SPSS Statistics, Inc., Chicago, IL).

## Results

### Study Population Characteristics

The study included 412 infants (229 boys and 183 girls) and their families (Table 1). In total, 9.9% of all infants were exposed to antibiotics prenatally and 32.5% were born by cesarean delivery. Moreover, 47.7% of the infants had at least one parent with a history of allergic diseases and 28.2% were diagnosed with AD at 12 months of age.

**Table 1:** Characteristics of the participating infants.

Individual characteristics	Number (n/d)*	Percentage (%) or mean ± SD
Male gender	229/412	55.6
Gestational age at birth, weeks		39.2±1.2
Maternal education level		
Low	300/393	76.3
High†	93/393	23.7
Mother’s age at delivery, years		32.3±3.5
Pre-pregnancy maternal body-mass index		20.8±2.7
Prenatal smoking		
No	150/384	39.1
Passive	234/384	60.9
Prenatal exposure to pets	11/381	2.9
Prenatal exposure to antibiotics	38/384	9.9
Mode of delivery		
Vaginal	210/311	67.5
Cesarean section	101/311	32.5
History of parental allergic diseases	178/373	47.7
Older sibling(s)	138/390	35.4
Atopic dermatitis ever	116/412	28.2

*n, number of children with each characteristic; d, the total number of children with information available on this characteristic.

† graduated from graduate school.

### Relationship between AD in Infancy and Delivery Mode and/or Prenatal Antibiotic Exposure

In multivariable regression analyses, cesarean section increased the odds or AD in infancy by 1.84 (95% CIs 0.95–3.56; p = 0.07) and prenatal antibiotic exposure did not increased (data not shown). The combination of cesarean section and prenatal antibiotic exposure significantly increased the odds of AD in infancy (adjusted OR [aOR] 5.70, 95% CIs 1.19–27.3, p = 0.03). Stool analysis at 6 months of age in 11 randomly selected infants revealed that the total microbiota of the infants with prenatal antibiotic exposure and cesarean delivery was less diverse than the total microbiota of the control infants (Fig. S1).

### Effect of Parental Allergic Disease History, Delivery Mode, and/or Prenatal Antibiotic Exposure on the Development of AD in Infancy

Table 2 and table S1 show how the development of AD in infancy related to cesarean section birth and/or prenatal antibiotic exposure when there was an allergic genetic background (defined as one or both parents having a history of allergic disease). In multivariable regression analyses, cesarean section significantly increased the odds of AD in infants when there was a history of parental allergic diseases (aOR 3.46, 95% CI 1.43–8.39, p<0.01, Table 2). Also, cesarean section significantly increased the odds of AD in infants than vaginal delivery in subjects with parental allergic history (aOR 2.83, 95% CI 1.03–7.73, p-value = 0.04, data not shown).

**Table 2:** Combined effects of mode of delivery and parental history of allergic diseases on the development of atopic dermatitis (AD) at 1 year of age.

Parental history of allergic diseases/Delivery mode	Number (%) of AD	OR (95% CI)	p-value	aOR (95% CI)*	p-value
Negative parental history/Vaginal delivery	25 (23.6)	1.00		1.00	
Negative parental history/C-section delivery	12 (24.5)	1.21 (0.59–2.50)	0.60	1.40 (0.57–3.47)	0.47
Positive parental history/Vaginal delivery	25 (30.9)	2.14 (1.19–3.83)	0.01	1.44 (0.69–3.01)	0.33
Positive parental history/C-section delivery	23 (48.9)	2.69 (1.36–5.29)	<0.01	3.46 (1.43–8.39)	<0.01

*Adjusted for gestational age at birth, sex, pre-pregnancy maternal body-mass index, maternal age at delivery, maternal education level, prenatal exposure to smoke, prenatal exposure to pets, and presence of older sibling(s).

However, prenatal antibiotic exposure did not significantly elevate the odds of AD in infants when there was a parental allergic disease history (Table S1).

When the three factors (parental allergic disease history, cesarean delivery, and prenatal antibiotic exposure) were each defined as risk factor of AD in infancy, multivariable regression analysis revealed that the more risk factors there were, the higher the likelihood that the infant would be diagnosed with AD at the age of 1 year (aOR 10.62, 95% CI 1.28–88.37, p = 0.03; Table 3).

**Table 3:** Additive effects of mode of delivery, prenatal antibiotic exposure, and parental history of allergic diseases on the development of atopic dermatitis (AD) at 1 year of age.

Number of risk factors†	Number (%) of AD	OR (95% CI)	p-value	aOR (95% CI)*	p-value
Risk (0)	20 (21.3)	1.00		1.00	
Risk (1)	35 (28.9)	1.51 (0.80–2.83)	0.20	1.83 (0.89–3.73)	0.10
Risk (2)	22 (42.3)	2.71 (1.30–5.68)	0.01	3.10 (1.28–7.51)	0.01
Risk (3)	5 (62.5)	6.17 (1.36–28.03)	0.02	10.62 (1.28–88.37)	0.03
Trend P-value			0.001		0.001

*Adjusted for gestational age at birth, sex, pre-pregnancy maternal body-mass index, maternal age at delivery, maternal education level, prenatal exposure to smoke, prenatal exposure to pets, and presence of older sibling(s).

† Risk factors were: parental history of allergic diseases, prenatal use of antibiotics, and cesarean delivery.

### Additive Effects between Cesarean Delivery or Prenatal Antibiotic Exposure and IL-13 or CD14 Gene Polymorphisms on Infant AD Risk

The distributions of two polymorphisms of the IL-13 and CD14 were in Hardy–Weinberg equilibrium. The genetic polymorphisms IL-13 rs20541 and CD14 rs2569190 themselves did not significantly influence the risk of AD in infancy (data not shown). However, compared to vaginally delivered infants with the IL-13 rs20541 GG genotype, vaginally delivered infants with the IL-13 rs20541 GA+AA genotypes tended to have a higher risk of infant AD (aOR 2.37, 95% CIs 0.96–5.84; p = 0.06; Table 4). The infants who had the GG genotype had a significantly higher risk of AD if they were delivered by cesarean section (aOR 3.85, 95% CI 1.25–11.83, p = 0.02). This effect of cesarean section was even more pronounced in infants who had the GA+AA genotypes (aOR 4.70, 95% CIs 1.43–15.40; p = 0.01).

**Table 4:** Combined effects of IL-13 or CD14 genetic variations and delivery mode on the development of atopic dermatitis (AD) at 1 year of age.

IL-13 rs20541/Delivery mode	Number (%) of AD	OR (95% CI)	p-value	aOR (95% CI)*	p-value
GG/Vaginal delivery	17 (22.7)	1.00		1.00	
GA+AA/Vaginal delivery	27 (26.7)	1.24 (0.61–2.54)	0.55	2.37 (0.96–5.84)	0.06
GG/C-section delivery	15 (45.5)	2.84 (1.19–6.81)	0.02	3.85 (1.25–11.83)	0.02
GA+AA/C-section delivery	13 (43.3)	2.61 (1.06–6.43)	0.04	4.70 (1.43–15.40)	0.01
**CD14 rs2569190/Delivery mode**					
TT/Vaginal delivery	13 (23.6)	1.00		1.00	
TC+CC/Vaginal delivery	28 (26.7)	1.18 (0.55–2.51)	0.68	1.34 (0.53–3.44)	0.54
TT/C-section delivery	9 (42.9)	2.42 (0.84–7.03)	0.10	3.11 (0.80–12.12)	0.10
TC+CC/C-section delivery	17 (43.6)	2.50 (1.03–6.06)	0.04	3.56 (1.12–11.36)	0.03

*Adjusted for gestational age at birth, sex, pre-pregnancy maternal body-mass index, maternal age at delivery, maternal education level, prenatal exposure to smoke, prenatal exposure to pets, presence of older sibling(s), and parental history of allergic diseases.

With regard to the CD14 genotypes, compared to vaginally delivered infants with the CD14 rs2569190 TT genotype, infants with the TT genotype who were born by cesarean section tended to have a higher risk of AD (aOR 3.11, 95% CIs 0.80–12.12, p = 0.10). This effect of cesarean section became significant in infants who had the CD14 rs2569190 TC+CC genotypes (aOR 3.56, 95% CI 1.12–11.36, p = 0.03; Table 3). Combined effect between CD14 rs2569190 and delivery mode has a significant trend P-value (P = 0.02).

With regard to prenatal antibiotic exposure, multivariable regression analysis revealed that there was a statistically significant trend for an association between this factor and IL-13 rs20541 genotype (trend p-value<0.05, data not shown). This trend was not observed for the CD14 rs2569190 genotype.

### Additive Effects between Unfavorable Genotypes and One or Two Environmental Risk Factors on the Development of AD in Infancy

**Figure 1:**
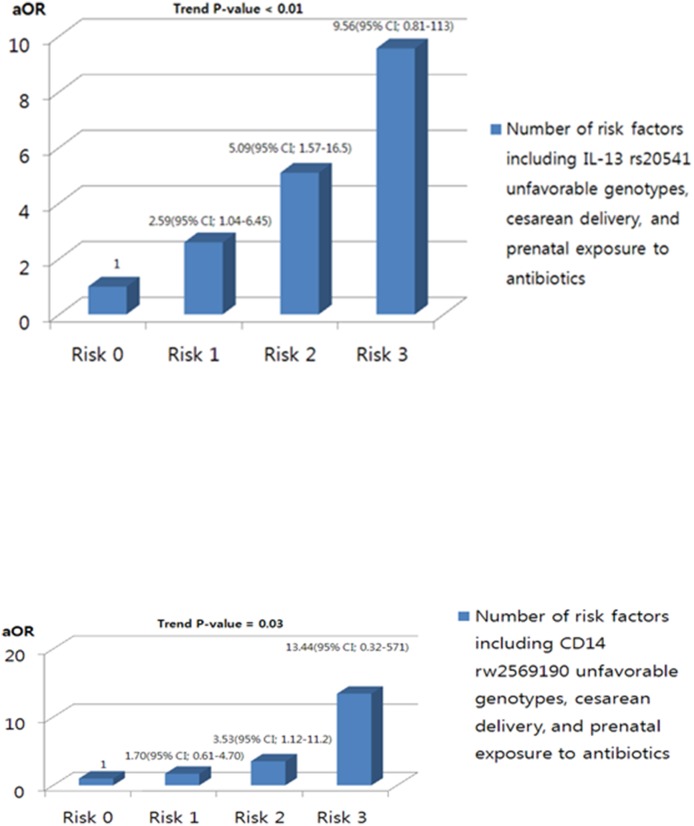
Additive effects of unfavorable IL-13 or CD14 genotypes, prenatal antibiotic exposure, and delivery mode on the development of atopic dermatitis in infancy. (A) IL-13 rs20541 genotype. (B) CD14 rs2569190 genotype. aOR, odds ratio adjusted for gestational age at birth, sex, pre-pregnancy maternal body-mass index, maternal age at delivery, maternal education level, prenatal exposure to smoke, prenatal exposure to pets, presence of older sibling(s), and parental history of allergic diseases.

The subjects were divided into four groups according to the number of risk factors, namely the presence of unfavorable IL-13 rs20541 or CD14 rs2569190 genotypes, prenatal antibiotic exposure, and cesarean delivery. The risk of AD in each group was then assessed relative to the group who had none of the three risk factors (Fig. 1 and Table S2). In terms of the IL-13 rs20541 genotype, the aOR for AD rose as the number of risk factors increased (one risk factor: aOR 2.59, 95% CIs 1.04–6.45; two risk factors: aOR 5.09, 95% CIs 1.57–16.52; three risk factors: aOR 9.56, 95% CIs 0.81–112.97; trend p-value<0.01). Although the statistical power to conduct analyses with the CD14 rs2569190 genotype was limited, the AD risk also rose as the number of risk factors increased (trend p-value 0.03, Table S2). Thus, unfavorable genotypes and exposure to prenatal environmental risk factors appeared act addictively in increasing AD risk in infancy.

## Discussion

This prospective birth cohort study showed for the first time that the impact of important intestinal microbiota-shaping risk factors (the delivery mode and prenatal antibiotic exposure) on the early development of AD can be modified by the genetic background (functional IL-13 and CD14 gene variants) in a additive fashion. The present study also showed that cesarean delivery and prenatal antibiotic exposure may affect the gut microbiota diversity in infants.

Although cesarean section and prenatal antibiotic exposure did not associate independently with an increased AD risk in infancy, infants who were delivered by cesarean section and had been exposed prenatally to antibiotics were more likely to have AD than those who were born vaginally and had not had prenatal antibiotic exposure. This trend was particularly notable in infants who carried risk-associated genotypes or whose parents had an allergic disease history. The present study confirms that the influence of cesarean section and prenatal antibiotic exposure on the development of AD should be assessed in the context of individual genetic susceptibility (e.g., parental allergic disease history). Another study reported a similar observation: Norwegian children who were born by cesarean section and had a maternal history of allergy had a 9-fold higher likelihood of food allergy than those who were born by vaginal delivery and had no maternal history of allergy [18].

In early life, the intestinal microbial composition changes markedly. A previous study showed that the mode of delivery significantly influences the microbiota composition of the intestine of neonates [19]. Interestingly, previous studies have also shown that the intestinal microbiota of cesarean section-born infants is similar to the microbiota of infants born to mothers who received antibiotics during late pregnancy and/or while breastfeeding [20], [21]. Other studies also reported that in cesarean delivered infants, there was a delay in fecal colonization and low numbers of *Bacteroides fragilis* [22], [23]. Moreover, in the first year of life, infants delivered by cesarean section do not catch up to vaginally delivered infants in terms of colonization by *Bacteroides* and *Escherichia coli* [24]. Hence, cesarean section and prenatal antibiotic exposure may promote the suboptimal development of the microbiota in infancy to similar extents. The present study showed that infants who had been exposed to antibiotics prenatally and were born by cesarean delivery had lower total microbiota diversity in the stool at 6 month of age than infants who did not have prenatal exposure to antibiotics and were born by vaginal delivery. The two groups did not differ significantly in terms of any of the dominant bacterial genera probably because of the small sample size. Thus, the findings of the present study support the notion that prenatal antibiotic exposure and delivery mode may affect the gut microbiota.

Although the gut microbiota is believed to play an intermediate role in the causal pathway between mode of delivery and AD, only one study has assessed this role directly [25]. That study showed that mode and place of delivery affect the gut microbiota at 1 month of age, especially in terms of *Clostridium difficile* colonization, and that this subsequently influences the risk of atopic manifestations at the age of 6–7 years. That study also showed that gut microbiota composition and mode and place of delivery both mainly associated with the development of allergic diseases in children with a positive family history of atopy. The present study demonstrated for the first time that not only cesarean delivery but also prenatal exposure to antibiotics play an important role in the development of AD in infancy, particularly in infants carrying IL-13 and CD14 genetic susceptibility alleles.

The present study showed that while cesarean section itself may influence the development of AD in infancy, this effect was enhanced in infants carrying the IL-13 rs20541 GA+AA genotypes. IL-13 rs20541 is a single nucleotide polymorphism (SNP) in the coding region of the IL-13 gene. In our previous study, children with the IL-13 GA+AA polymorphisms were found to be at increased risk of allergic rhinitis if they were exposed to mold in the home during their first year of life [26], and were associated with susceptibility of asthma [27]. For this reason, and because the combined effects between cesarean section or prenatal antibiotic exposure and IL-13 SNPs had not yet been examined, the IL-13 rs20541 SNP was selected for our study.

The present study also supports the notion that the C allele of the CD14 SNP rs2569190 confers a risk of AD in infants who are born by cesarean section. In rodents, oral exposure to lipopolysaccharides during vaginal birth activates gut epithelial cells [28]. By contrast, gut epithelial activation is not found in mice born by cesarean section. The Allergy and Endotoxin (ALEX) Study [29] supported the endotoxin switch theory that the C allele of CD14 rs2569190 confers risk at low endotoxin exposure whereas the T allele confers risk at high endotoxin exposure. Thus, infants with the C allele of CD14 SNP rs2569190 may be at higher risk of AD if their endotoxin exposure is low because their microbiota is undeveloped due to cesarean delivery and prenatal antibiotic exposure.

The strengths of the present study include its prospective nature and its population-based design. This is the first study to assess how cesarean section, prenatal antibiotic exposure, and gene susceptibility alleles interact in the development of AD in infancy while adjusting for other prenatal environment factors. We acknowledge that it also has some limitations. The main limitations are the relatively smaller sample size compared to those in similar studies. Our findings should be confirmed by larger studies. In addition, this study focused on only two well-known candidate genes that are involved in Th1/Th2 balance. It remains likely that other, as-yet unknown but important, genes can also regulate the influence of cesarean section and prenatal antibiotic exposure on the development of AD in infancy. However, IL-13 and CD14 genes are good candidate genes in terms of allergic susceptibility and innate immunity since several studies have shown that IL-13 rs1295685 and CD14 rs20541 play an important role in the development of allergic diseases in Korean children [26], [27], [30]. Another possible limitation is that the diet history of the infants in this study was not considered. As a result, the present study focused largely on the prenatal environmental factors that may influence the development of AD in infancy. We showed the limited results of difference of gut microbiota in small population because it is very difficult to find the pregnant women who had used antibiotics during pregnancy.

## Conclusions

We found that cesarean section, prenatal antibiotic exposure, and unfavorable risk alleles acted additively to promote the development of AD in infancy. The present findings suggest that further studies that evaluate the effect of gut microbiota on AD in the context of individual genetic variation are warranted. The type of information yielded by such studies might enable physicians in the future to provide more personalized medical care and advice to mothers that will reduce the risk of AD in their children.

## Supporting Information

File S1 **Supporting tables and figure.**
(DOCX)
