# Supplementary material for: Additive Effect between IL-13 Polymorphism and Cesarean Section Delivery/Prenatal Antibiotics Use on Atopic Dermatitis: A Birth Cohort Study (COCOA)
Source: PLoS One. 2014 May 21;9(5):e96603. doi: 10.1371/journal.pone.0096603 (PMC4029558; doi:10.1371/journal.pone.0096603)
Supplement: File S1 — Supporting tables and figure. (DOCX) [file pone.0096603.s001.docx]

Table S1. Combined effects of prenatal antibiotic exposure and parental history of allergic diseases on the development of atopic dermatitis (AD) at 1 year of age

| **Parental history of allergic diseases/Prenatal exposure to antibiotics (PEA)^†^** | **Number (%) of AD** | **OR (95% CI)** | **p-value** | **aOR (95% CI)*** | **p-value** |
| --- | --- | --- | --- | --- | --- |
| Negative parental history/Negative PEA | 39 (22.2) | 1.00 |  | 1.00 |  |
| Negative parental history/Positive PEA | 6 (42.9) | 2.64 (0.86–8.05) | 0.09 | 2.73 (0.80–9.30) | 0.11 |
| Positive parental history/Negative PEA | 49 (32.5) | 1.69 (1.03–2.76) | 0.04 | 1.58 (0.88–2.80) | 0.12 |
| Positive parental history  /Positive PEA | 9 (40.9) | 2.43 (0.97–6.11) | 0.06 | 2.11 (0.63–7.07) | 0.23 |

* Adjusted for gestational age at birth, sex, pre-pregnancy maternal body-mass index, maternal age at delivery, maternal education level, prenatal exposure to smoke, prenatal exposure to pets, and presence of older sibling(s).

^†^ PEA, Prenatal exposure to antibiotics

Table S2. Additive effects between IL-13 or CD14 genetic polymorphism, prenatal antibiotic exposure, and delivery mode on the development of atopic dermatitis (AD) at 1 year of age

| **Number of risk factors^†^** | **Number (%) of AD** | **OR (95% CI)** | **p-value** | **aOR (95% CI)*** | **p-value** |
| --- | --- | --- | --- | --- | --- |
| **IL-13 rs20541** |  |  |  |  |  |
| Risk (0) | 11 (19.6) | 1.00 |  | 1.00 |  |
| Risk (1) | 35 (30.7) | 1.81 (0.84–3.91) | 0.13 | 2.59 (1.04–6.45) | 0.04 |
| Risk (2) | 13 (39.4) | 2.66 (1.02–6.95) | 0.05 | 5.09 (1.57–16.52) | <0.01 |
| Risk (3) | 3 (60.0) | 6.14 (0.91–41.3) | 0.06 | 9.56 (0.81–112.97) | 0.07 |
| Trend P-value |  |  | 0.01 |  | <0.01 |
| * Adjusted for gestational age at birth, sex, pre-pregnancy maternal body-mass index, maternal age at delivery, maternal education level, prenatal exposure to smoke, prenatal exposure to pets, presence of older siblings, and parental history of allergic diseases.  ^†^ The risk factors are: unfavorable alleles of each gene, prenatal antibiotic use, and cesarean delivery. | | | | | |
| **CD14 rs2569190** |  |  |  |  |  |
| Risk (0) | 9 (22.0) | 1.00 |  | 1.00 |  |
| Risk (1) | 30 (27.3) | 1.33 (0.57–3.12) | 0.51 | 1.70 (0.61–4.70) | 0.31 |
| Risk (2) | 21 (43.8) | 2.77 (1.09–7.04) | 0.03 | 3.53 (1.12–11.17) | 0.03 |
| Risk (3) | 1 (50.0) | 3.56 (0.20–62.6) | 0.39 | 13.44 (0.32–571.18) | 0.17 |
| Trend P-value |  |  | 0.02 |  | 0.02 |

* Adjusted for gestational age at birth, sex, pre-pregnancy maternal body-mass index, maternal age at delivery, maternal education level, prenatal exposure to smoke, prenatal exposure to pets, presence of older siblings, and parental history of allergic diseases.

^†^ The risk factors are: unfavorable alleles of each gene, prenatal antibiotic use, and cesarean delivery.

Figure S1. Diversity of the total microbiota in stool samples obtained at 6 months of age from five infants who were born by cesarean delivery and were exposed prenatally to antibiotics (case) and six infants who were born by vaginal delivery and did not have a history of prenatal exposure to antibiotics (control). The distribution of bacterial diversity (A) and phylum compositions (B) in each case and control are shown.

1.
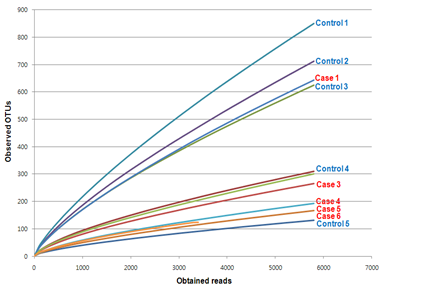


OTUs : Operational Taxonomic Units


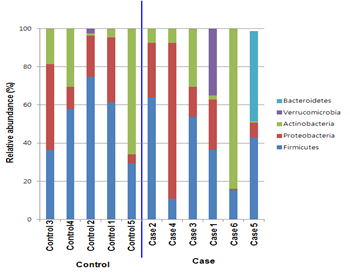


**Supporting Information Legends**

Table S1. Combined effects of prenatal antibiotic exposure and parental history of allergic diseases on the development of atopic dermatitis at 1 year of age

Table S2. Additive effects between IL-13 or CD14 genetic polymorphism, prenatal antibiotic exposure, and delivery mode on the development of atopic dermatitis in infancy

**Figure S1. Diversity of the total microbiota in stool samples.**

It obtained at 6 months of age from five infants who were born by cesarean delivery and were exposed prenatally to antibiotics (case) and six infants who were born by vaginal delivery and did not have a history of prenatal exposure to antibiotics (control). The distribution of bacterial diversity (A) and phylum compositions (B) in each case and control are shown.
